# Fetal lung growth predicts the risk for early-life respiratory infections and childhood asthma

**DOI:** 10.1007/s12519-023-00782-y

**Published:** 2024-01-23

**Authors:** Dimitra E. Zazara, Olympia Giannou, Steven Schepanski, Mirja Pagenkemper, Anastasios D. Giannou, Maike Pincus, Ioannis Belios, Stefan Bonn, Ania C. Muntau, Kurt Hecher, Anke Diemert, Petra Clara Arck

**Affiliations:** 1 Division for Experimental Feto-Maternal Medicine, Department of Obstetrics and Fetal Medicine, University Medical Center Hamburg-Eppendorf (UKE), Martinistraße 52, 20251 Hamburg, Germany; 2 University Children’s Hospital, UKE, Hamburg, Germany; 3 Computer Engineering and Informatics Department, Polytechnic School, University of Patras, Patras, Greece; 4 Institute of Developmental Neurophysiology, Center for Molecular Neurobiology Hamburg (ZMNH), UKE, Hamburg, Germany; 5 Department of Obstetrics and Fetal Medicine, UKE, Hamburg, Germany; 6 Department of General, Visceral and Thoracic Surgery, UKE, Hamburg, Germany; 7 Section of Molecular Immunology and Gastroenterology, I. Department of Medicine, UKE, Hamburg, Germany; 8 Pediatrics and Pediatric Pneumology Practice, Berlin, Germany; 9 Institute of Medical Systems Biology, ZMNH, UKE, Hamburg, Germany; 10 Hamburg Center for Translational Immunology, UKE, Hamburg, Germany

**Keywords:** Asthma, Child risk, Early-life respiratory infections, Prenatal lung development, Sexual dimorphism

## Abstract

**Background:**

Early-life respiratory infections and asthma are major health burdens during childhood. Markers predicting an increased risk for early-life respiratory diseases are sparse. Here, we identified the predictive value of ultrasound-monitored fetal lung growth for the risk of early-life respiratory infections and asthma.

**Methods:**

Fetal lung size was serially assessed at standardized time points by transabdominal ultrasound in pregnant women participating in a pregnancy cohort. Correlations between fetal lung growth and respiratory infections in infancy or early-onset asthma at five years were examined. Machine-learning models relying on extreme gradient boosting regressor or classifier algorithms were developed to predict respiratory infection or asthma risk based on fetal lung growth. For model development and validation, study participants were randomly divided into a training and a testing group, respectively, by the employed algorithm.

**Results:**

Enhanced fetal lung growth throughout pregnancy predicted a lower early-life respiratory infection risk. Male sex was associated with a higher risk for respiratory infections in infancy. Fetal lung growth could also predict the risk of asthma at five years of age. We designed three machine-learning models to predict the risk and number of infections in infancy as well as the risk of early-onset asthma. The models’ *R*^2^ values were 0.92, 0.90 and 0.93, respectively, underscoring a high accuracy and agreement between the actual and predicted values. Influential variables included known risk factors and novel predictors, such as ultrasound-monitored fetal lung growth.

**Conclusion:**

Sonographic monitoring of fetal lung growth allows to predict the risk for early-life respiratory infections and asthma.

**Graphical abstract:**

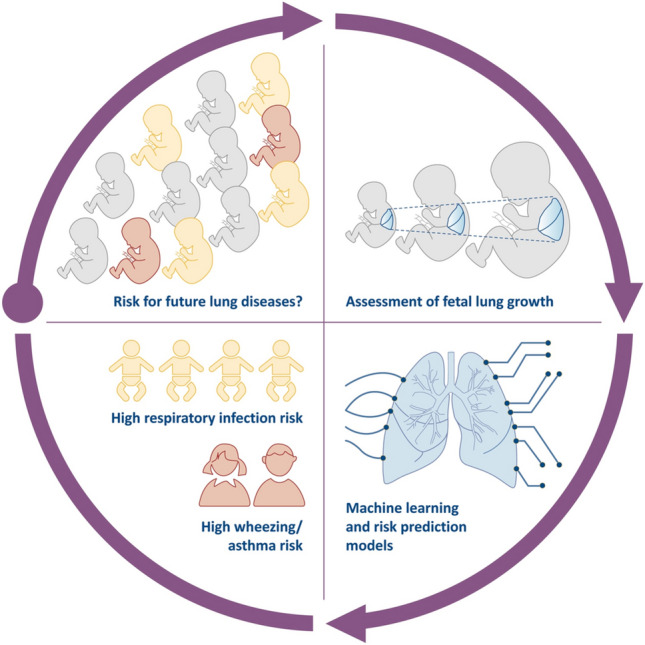

**Supplementary Information:**

The online version contains supplementary material available at 10.1007/s12519-023-00782-y.

## Introduction

Respiratory diseases such as infections and allergic asthma are major causes of morbidity and mortality in neonates and children [1–3]. Acute respiratory tract infections, especially those involving the upper airways, are the most common illnesses in young children [4, 5]. Given that they are usually treated in outpatient settings, the exact incidence of upper respiratory tract infections is often hard to determine, and most epidemiological studies provide information collected in hospital settings and thus refer to the typically more severe lower respiratory tract infections [6–8]. According to the Global Burden of Diseases, Injury and Risk Factors Study 2015, lower respiratory tract infections are the third leading cause of death in children younger than 5 years in 195 countries worldwide, resulting in 12.1% of deaths in this population [1]. Epidemiological data highlight the predominance of viruses in childhood respiratory tract infections. Specifically, 90% of upper respiratory tract infections are of viral origin, with the most common pathogens being rhino- and adenovirus [4]. Lower respiratory tract infections are attributed to viruses in approximately 50% of cases, with respiratory syncytial virus (RSV), adenovirus, metapneumovirus, influenza, and parainfluenza most frequently causing the disease [4, 9, 10].

Similarly, asthma is the most common non-communicable disease in children [11, 12], with an uprecedented worldwide incidence of nearly 22 million childhood cases in 2019 [13]. The burden of childhood asthma also becomes evident from the rate of morbidity and mortality, e.g., 12,900 children died from asthma, and 5.1 million disability-adjusted life years were associated with asthma in 2019 worldwide [13]. Epidemiological studies not only highlight the increasing incidence of these diseases worldwide but also demonstrate a considerable association between early-life infections and the increased risk of subsequently developing childhood asthma [14–18].

Fetal and early-life lung development pave the way for lung function and pathology later in life. Lung development is a delicate process consisting of distinct pre-, peri- and postnatal events that determine lung function throughout life [19]. An underdeveloped lung structure and function, e.g., seen in premature-born children, increases the risk for respiratory diseases, such as early-life infections and wheezing disorders during childhood [20, 21].

Despite these intriguing epidemiological and developmental insights, studies monitoring features of fetal lung growth and its association with postnatal respiratory health are missing. To close this critical gap in knowledge, we took advantage of the availability of highly granular data from a prospectively designed observational pregnancy study, focusing mainly on uncomplicated pregnancies and term-born offspring. Here, fetal lung development could be evaluated using scans from serially acquired ultrasound examinations throughout gestation and subsequently linked to respiratory health or diseases recorded during infancy and childhood. In our study, we combined serial monitoring of fetal lung growth trajectories with a machine-learning approach and developed accurate models to identify children at risk for respiratory diseases.

## Methods

### Study design

The present work was conducted within the Prenatal Identification of Children’s Health (PRINCE) study. The PRINCE study is a prospective longitudinal pregnancy cohort located at the University Medical Center Hamburg-Eppendorf, which started in 2011 and focuses on the impact of prenatal challenges on children’s health. The inclusion criteria for pregnant women to enrol were an age ≥ 18 years and a viable singleton pregnancy of 12–14 weeks of gestation. Exclusion criteria included chronic infections (human immunodeficiency virus, hepatitis B or C), known drug or alcohol abuse, multiple pregnancies or pregnancies resulting from assisted reproductive technology. Pregnancy progression, health status, medication, stress perception, and anthropometric data of the mother were documented during study visits between 12 and 14, 24 and 26, and 34 and 36 weeks of gestation. Transabdominal ultrasound examinations were also performed at these study visits. At a gestational age of 27–29 weeks, study participants were offered the opportunity for an additional ultrasound assessment as part of their prenatal visit at our hospital to register for giving birth.

**Fig. 1:**
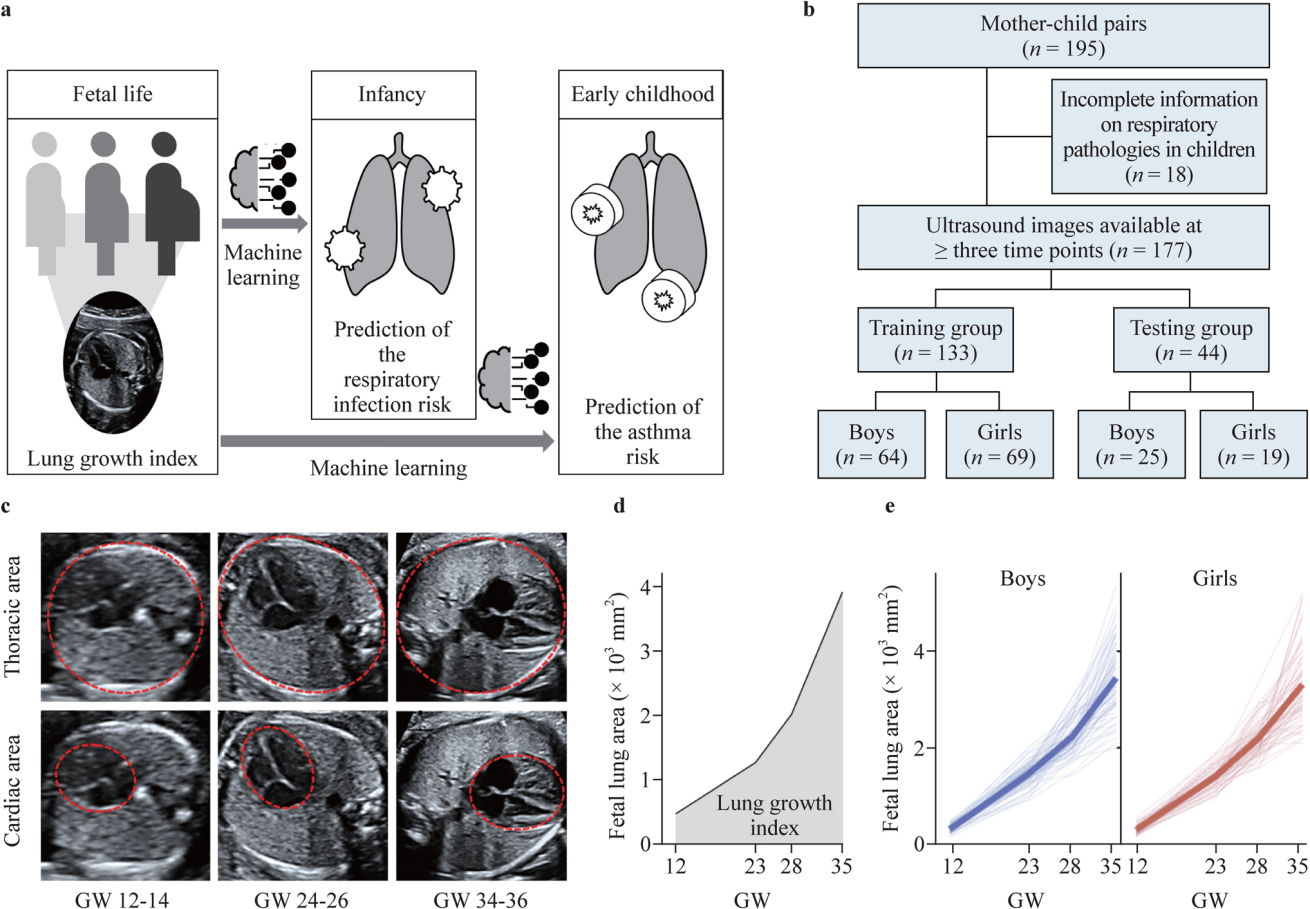
Using ultrasound to monitor the progress of fetal lung growth. **a** Graphical presentation of the study timeline; **b** flow chart of the study participants in the training and testing groups; **c** fetal ultrasound pictures at the four-chamber view of the fetal heart acquired in gestational weeks (GW) 12–14, 24–26, and 34–36 (in red the thoracic and cardiac areas); **d** graph showing the lung growth index (gray area); **e** fetal lung growth trajectories for boys and girls (the thick line represents the mean trajectory for each group)

At birth, anthropometric indices of the newborn were obtained. At the age of twelve months (infancy), information on the occurrence of upper and lower respiratory tract infections was obtained by standardized parental questionnaires and independently confirmed by the routine childhood screenings of the child’s pediatrician. Specifically, the documented infections were common cold, pneumonia, tonsillitis, bronchitis and croup. The health status of the children was followed up annually between the ages of two and four years. At the age of five years, a study visit of the child was performed by a trained pediatrician (Fig. 1a). From the mother/child pairs that had participated in pre- and postnatal study visits of the study until the children’s age of five years by March 2021 (*n* = 195), prenatal and postnatal datasets were available for 177 mother/child pairs. From these, 75% (*n* = 133) of the total available pairs were randomly selected by the employed algorithm and included in the current analysis as a training group to develop (train) three models to predict the exact number and risk of respiratory infections in infancy as well as the risk for early-life asthma manifestation on the basis of the ultrasound-monitored fetal lung growth trajectory, while the remaining independent participant group (*n* = 44; 25% of total pairs) served as a testing group to conclusively validate the predictive value of the developed models (Fig. 1b). Mother/child pairs included in either cohort are referred to as study participants throughout the manuscript.

### Study approval

The study protocol of the PRINCE study was approved by the Ethics Committee of the Hamburg Chamber of Physicians under the registration number PV3694 and performed in compliance with the Declaration of Helsinki for Medical Research involving Human Subjects. Informed consent forms to participate in the study were signed by all participants (or their parent or legal guardian in the case of children under 16).

### Ultrasound measurements

Ultrasound examinations and measurements were performed using a Voluson E8 (General Electric; GE) equipped with a transabdominal 3–5 MHz transducer (RAB 6D, GE). Measurements were conducted by two trained clinicians with certified advanced ultrasound expertise. A routine standard sonographic study, including fetal biometry and anomaly screening, was always included. Based on the study design, estimated fetal weight was calculated using the Warsof formula in the first trimester and the Hadlock IV formula at the other two time points [22, 23]. The lung area was calculated by subtracting the heart area from the thorax at the four-chamber level and averaging three replicate values at each time point [24]. Ultrasound examinations that precluded fetal lung measurements due to fetal position or fetal movements affected approximately 11% of all cases and were excluded.

### Infection and asthma risk classification

Based on the number of respiratory infections in infancy, two groups with distinct infection risks could be identified. Specifically, children who exhibited five or fewer respiratory infections in the first year of life were identified as being at low infection risk, whereas a high risk for infection characterized children with a history of at least six respiratory infections in infancy [25]. The classification of the risk for early-onset asthma was based on clinical information and examination performed by a pediatrician at the age of five years. In addition to the clinical examination, the physician employed standardized clinical information and criteria that have been developed and universally applied by several population-based birth cohort studies focusing on asthma and have been validated in the International Study of Asthma and Allergies in Childhood project worldwide [26, 27]. Specifically, the current existence of an asthmatic phenotype was affirmative upon fulfilment of two out of the three following criteria: (1) pre-existing asthma diagnosis by a physician; (2) any asthma-related symptom within the last 12 months, including wheezing, dry cough at night and shortness of breath; and (3) use of asthma medication within the last 12 months [27]. As a complementary approach, a second classification system based on guidelines that have been established and validated by German health authorities with the special aim of identifying two- to five-year-old children with early signs of an asthmatic predisposition was also used [28]. Specifically, a high risk for early-onset asthma development was present in children who exhibited three asthma-typical episodes in the last 12 months that responded well to asthma medication and satisfied at least one of the following criteria: (1) existence of a parent or sibling suffering from asthma; (2) hospitalization due to obstructive respiratory symptoms; (3) clinical evidence of sensitization; and (4) wheezing without an underlying respiratory infection. Children with a positive scoring outcome in either one scoring approach were considered to be at high risk for early-onset asthma development.

### Statistical analysis

#### General statistics

Study sample characteristics, including demographics and ultrasound parameters, are presented as the mean ± standard deviation. Comparison of the maternal and neonatal demographic parameters between the training and testing cohorts was performed by using the Chi-squared test or the Mann‒Whitney *U* test with a significance level of *P* < 0.05. Comparison of the number of infections per child between sexes was performed using the Mann‒Whitney *U* test. The data shown represent the mean ± standard error of the mean. The respective analysis was conducted, and plots were created with GraphPad Prism, version 8.0 (GraphPad Software, Inc., La Jolla, California), R version 4.1.2 and Python scripting language version 3.8.

#### Missing fetal lung growth values and design of the fetal lung growth trajectory

To design the trajectory of fetal lung growth covering the entire prenatal period of interest for all male and female fetuses, values for common standardized time points, namely, gestational weeks 12, 23, 28 and 35, for all children were needed. For this reason, missing values for any standardized time points, e.g., ultrasound examination conditions that did not allow fetal lung area assessment (as mentioned above), were imputed using Poisson regression based on the available fetal lung area measurements for each fetus. The imputed data and related fetal lung growth trajectories were used for the calculation of the area under the curve by applying the linear trapezoidal rule [29]. The estimated area under the curve was subsequently used as a representative summarizing indicator for each child’s fetal lung growth trajectory for further prediction analysis and risk assessment and is referred to as the “lung growth index” throughout the manuscript.

#### Regression analysis

A Poisson regression model with log as the link function was used to analyze the influence of fetal growth and fetal lung growth index, maternal age at birth, maternal smoking during pregnancy, maternal body mass index (BMI) in the first trimester, mean maternal stress perception during pregnancy, gestational age and weight of the offspring at birth, child sex, and the presence of older siblings on the number of respiratory infections during the first year of life [30]. This model was further adjusted by using the natural logarithm of the time interval in gestational weeks between the minimum and the maximum available fetal lung growth value for each child as an offset.

#### Machine learning approach to predict the number of respiratory infections in infants

To predict the total number of respiratory infections in the first year of life (model I), machine learning modeling was used. The lung growth index during pregnancy, gestational age and weight of the offspring at birth, child sex, maternal first trimester BMI, and age at birth were the included input data features. Four types of regression algorithms, namely, K-nearest neighbors regressor (KNNR) [31], random forest regressor (RFR) [32], gradient boosting regressor (GBR) [33], and extreme gradient boosting regressor (XGBR) [34], were implemented to determine the optimal strategy and prediction model. All regressor models were evaluated on our dataset using tenfold cross validation, based on the following metrics [35]: (1) *R*-squared (*R*^2^), the correlation coefficient representing how well the model fits, i.e., the closer this value is to 1, the more perfectly the model performs; (2) mean squared error, demonstrating the difference between the actual observations and the observation values predicted by the model; (3) root mean squared error, measuring the average difference between predicted by the model and actual values; and (4) mean absolute error (MAE), the absolute difference between the actual value and the one predicted by the model, i.e., the lower the MAE, the better the model. To determine the impact of each input feature on the prediction, Shapley additive explanations (SHAP) values were used [36].

#### Machine learning approach to predict the risk for respiratory infections in infancy or asthma manifestation in early childhood

Machine learning approaches were again employed to develop an additional model predicting the risk (low or high) for respiratory infections in infancy (model II) and a third model predicting the risk for early-onset childhood asthma development (model III). Similar to model I mentioned above, the input data features included the lung growth index during pregnancy, gestational age and weight of the offspring at birth, child sex, maternal first trimester BMI, and age at birth. In this case, and due to our experience with the abovementioned modeling strategy, the algorithm that we used for prediction was the XGBC [34]. The confusion matrix, accuracy, precision, recall (also known as true positive rate or sensitivity), f1 score, and the receiver operating characteristic (ROC) curve along with the area under the ROC curve were used to evaluate the diagnostic performance of each model [37, 38].

## Results

### Study participant demographics and characteristics

The demographics and characteristics of the study participants included in the training and testing groups are shown in Table 1. Apart from parity, no significant differences were present between the training and testing groups with regard to demographic, anthropometric, and educational parameters in mothers or neonates [39]. This includes parameters such as advanced maternal age (≥ 35 years) [40] and grand multiparity (≥ 5 pregnancies resulting in viable offspring) [41] in the training and testing groups. According to the exclusion criteria described earlier, multiple pregnancies or pregnancies resulting from assisted reproductive technology were excluded from the study. Among the 177 children included in the training and testing groups, one child was diagnosed with intrauterine growth restriction (IUGR). In this child, the lung growth was indeed below average (data not shown), as expected, but exclusion did not affect the overall outcome, and it was thus included in the study. Along this line, we wish to highlight that our aim was to predict the risk for childhood infection and asthma based on lung growth, rather than focusing on such risk in distinct subgroups, such as children born upon pregnancy complications or IUGR. Fetal ultrasound assessment and lung area measurement were performed at three main time points during pregnancy (Fig. 1c). To assess the quality and accuracy of the obtained lung ultrasound measurements, the intraclass correlation coefficient (“one-way” model; “agreement” type) was calculated in samples of the training group and revealed excellent agreement for ultrasound assessments at all time points of interest between two independent blinded observers (Supplementary Fig. 1a). Mean ultrasound parameters throughout pregnancy for the training and testing groups are shown in Supplementary Tables 1 and 2, respectively. To design the fetal lung growth trajectory throughout pregnancy based on standardized time points, data imputation was performed. Importantly, the calculation of Pearson’s correlation coefficient showed excellent agreement between the actual and predicted values for the fetal lung area (Supplementary Fig. 1b). Subsequently, fetal lung growth trajectories were designed, and their progress was quantified based on the estimated area under the curve, here referred to as the lung growth index (Fig. 1d and e). To exclude potential aberrations in fetal growth, trajectories depicting the fetal growth course were also designed based on the estimated fetal weight at the three main time points. In this case, the estimated area under the curve served as an indicator of fetal growth throughout pregnancy (Supplementary Fig. 1c).

**Table 1:** Characteristics of the training and testing groups as well as the entire study cohort (*N* = 660) [75]

Variables	Training group (*n* = 133)		Testing group (*n* = 44)		P^*^	Entire cohort
	Boys (*n* = 64)	Girls (*n* = 69)	Boys (*n* = 25)	Girls (*n* = 19)		
Maternal parameters						
Age at birth (y), mean ± SD^a^	32.4 ± 3.4	32.6 ± 4.1	32.6 ± 4.0	33.6 ± 3.9	0.704	31.9 ± 3.7
Parity, mean ± SD^b^	1.3 ± 0.5	1.5 ± 0.7	1.6 ± 0.6	1.6 ± 0.7	0.010	1.7 ± 1.0
First trimester BMI (kg/m^2^), mean ± SD	24.1 ± 3.5	24.0 ± 3.6	25.1 ± 5.0	25.7 ± 5.1	0.301	24.2 ± 4.0
Underweight (< 18.5 kg/m^2^), %	0.0	2.9	0.0	0.0		1.6
Normal weight (18.5–24.9 kg/m^2^), %	64.1	63.8	72.0	57.9		66.5
Overweight (≥ 25 kg/m^2^), %	35.9	33.3	28.0	42.1		31.9
Educational level, %					0.433	
School attendance ≤ 10 y	24.6	19.1	32.0	26.3		22.2
School attendance 10–13 y	27.9	35.3	28.0	42.1		27.9
University degree	47.5	45.6	40.0	31.6		47.1
Neonatal parameters						
GW, mean ± SD	39.1 ± 1.2	38.8 ± 1.6	39.3 ± 1.2	39.0 ± 1.4	0.787	39.0 ± 1.6
Preterm (GA < 37 wk), %	1.6	8.7	0.0	0.0	0.197	5.5
Birth weight (g), mean ± SD	3499.4 ± 536.4	3412.3 ± 516.5	3648.6 ± 446.7	3393.8 ± 513.4	0.349	3472.2 ± 480.7
Height at birth (cm), mean ± SD	52.1 ± 2.2	51.6 ± 2.3	52.4 ± 2.2	51.1 ± 2.7	0.921	51.9 ± 2.4
Sex, %	48.1	51.9	56.8	43.2	0.385	51.4 (males)/48.6 (females)

*BMI* body mass index, *GW* gestational weeks, *GA* gestational age, *SD* standard deviation. ^a^Advanced maternal age (≥ 35 years) characterized 29% and 30% of the mothers included in the training and testing groups, respectively; ^b^in total, one out of the 177 (0.6%) mothers included in the study was grand multipara. ^*^Comparison between the training and testing groups, independent of sex, was performed by using the Chi-squared or the Mann–Whitney *U* test, as appropriate, with a significant level at *P* < 0.05

### Respiratory infections in infancy and asthma manifestation in childhood

**Fig. 2:**
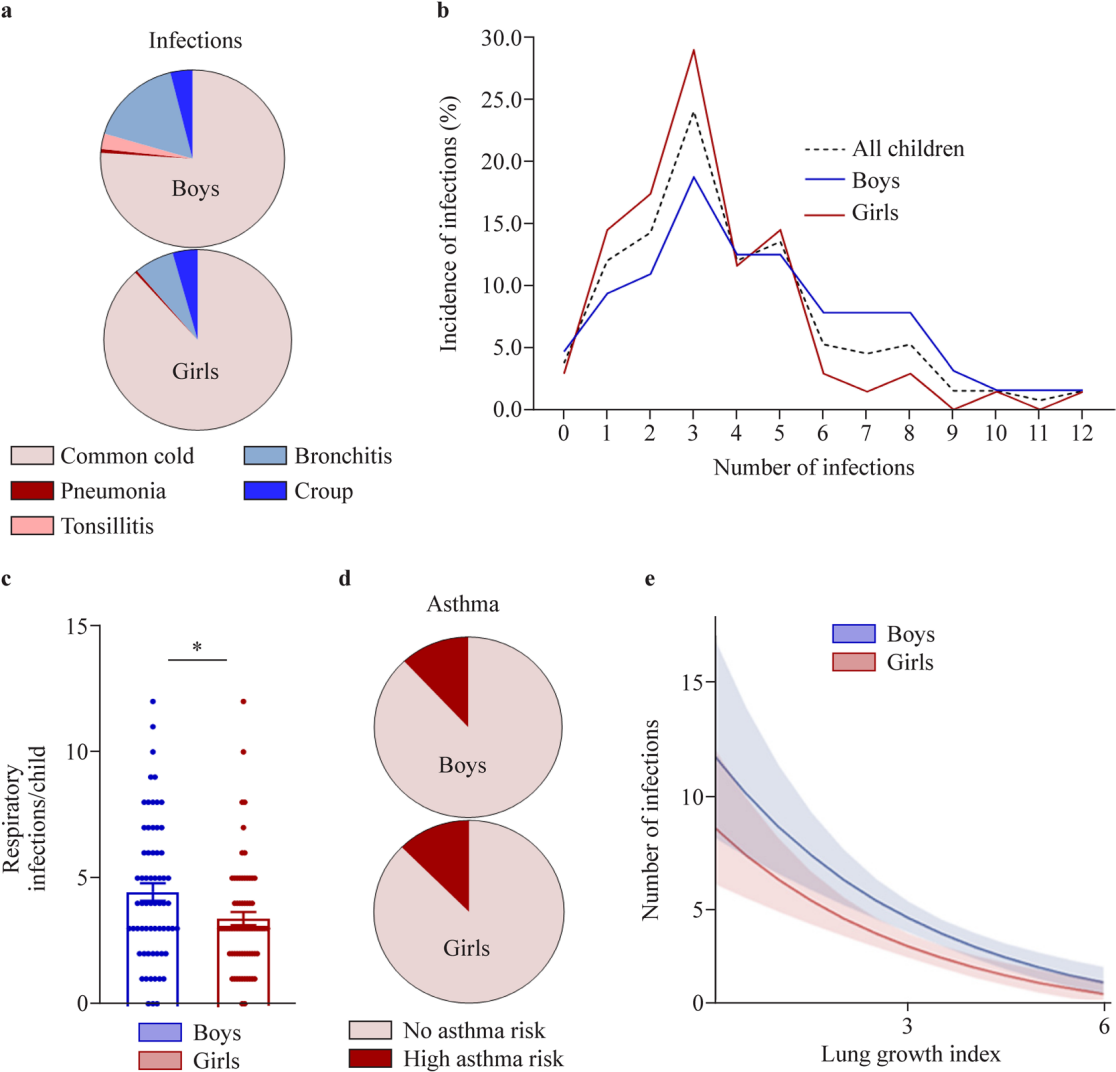
Respiratory infections in infancy and risk for asthma early in childhood. **a** Documented respiratory infections and their prevalence in boys and girls of the training group; **b** infection count in infancy expressed as a percentage of all boys, girls and all children in the training group; **c** respiratory infections per child; **d** risk for asthma in boys and girls of the training group; **e** graphic depiction of the Poisson regression model showing the impact of fetal lung growth on the risk for respiratory infections (bars represent the mean ± standard error of mean). ^*^P ≤ 0.05 as assessed by Mann‒Whitney *U* test

During the first year, the common cold was the most frequent respiratory infection in both boys and girls (Fig. 2a; Supplementary Tables 3 and 4). Among the 133 children included in the training group, five (3.76%) children exhibited no respiratory infections in the first year of life, while the majority (24%) of children suffered from three respiratory infections during this time. In general, most children were classified as being at low infection risk, while the minority exhibited more than six respiratory infections and were characterized by a high infection risk. The highest documented number of respiratory infections until the first birthday was twelve (Fig. 2b). Although the total number of infections did not differ between boys and girls, the mean number of infections in boys was significantly higher than that in girls (Fig. 2c). Notably, testing for specific pathogens causing a respiratory infection is not routinely performed in our country (and many other countries), unless the course of the infection is very severe and the clinical symptoms require additional attention, such as hospitalization. Hence, among the total number of children included in the training and testing groups (*n* = 177), polymerase chain reaction-based testing of the pathogen causing the respiratory infection was available only in five children, four of which tested positive for RSV infection and one child for influenza A virus. Regarding early-onset asthma, a positive asthma risk classification was identified in 12.23% of all participating children at the age of 5 years, based on at least one of the applied scoring systems, with boys and girls being similarly affected (Fig. 2d).

### Fetal lung growth as a predictor for early-life respiratory morbidities

Using a Poisson regression model, we next aimed to identify risk predictors for respiratory infections in infancy. Among all examined factors, fetal lung growth, here evident as the lung growth index, as well as the sex of the offspring were found to significantly affect the risk for early-life respiratory infections. Importantly, an enhanced progress of fetal lung growth, specifically a one-point increase in the lung growth index, would result in a decrease in the respiratory infection risk ratio by a factor of 0.78, while holding all other variables in the model constant (Fig. 2e; Table 2). Additionally, male sex was identified as an independent risk factor for early-life respiratory infections if all other parameters in the model were constant (Table 2), meaning also that, among children with a similar lung growth index, boys are at higher risk for suffering from frequent respiratory infections in infancy.

**Table 2:** Impact of prenatal and postnatal parameters on the risk for early-life respiratory infections as calculated using a Poisson regression model

Predictors	Total infection count		
	Risk ratio	95% Cl	*P*
Intercept	0.43	0.02–10.4	0.610
Lung growth index (mm^2^)	0.73	0.66–0.81	< 0.001
Fetal growth (kg)	1.26	0.86–1.88	0.241
Maternal age	1.00	0.97–1.02	0.777
Maternal smoking (yes)	1.09	0.77–1.54	0.630
Maternal first trimester BMI (kg/m^2^)	1.01	0.99–1.04	0.293
Maternal stress perception (mean PSS-10)	1.01	1.00–1.03	0.321
Gestational age at birth (wk)	0.95	0.87–1.03	0.223
Birth weight (kg)	1.00	0.99–1.00	0.080
Sex (male)	1.36	1.14–1.64	0.001
Older siblings (yes)	1.06	0.88–1.06	0.533

*CI* confidence interval, *BMI* body mass index, *PSS-10* perceived stress scale-10

### Using machine learning to predict susceptibility to respiratory infections in infancy

**Fig. 3:**
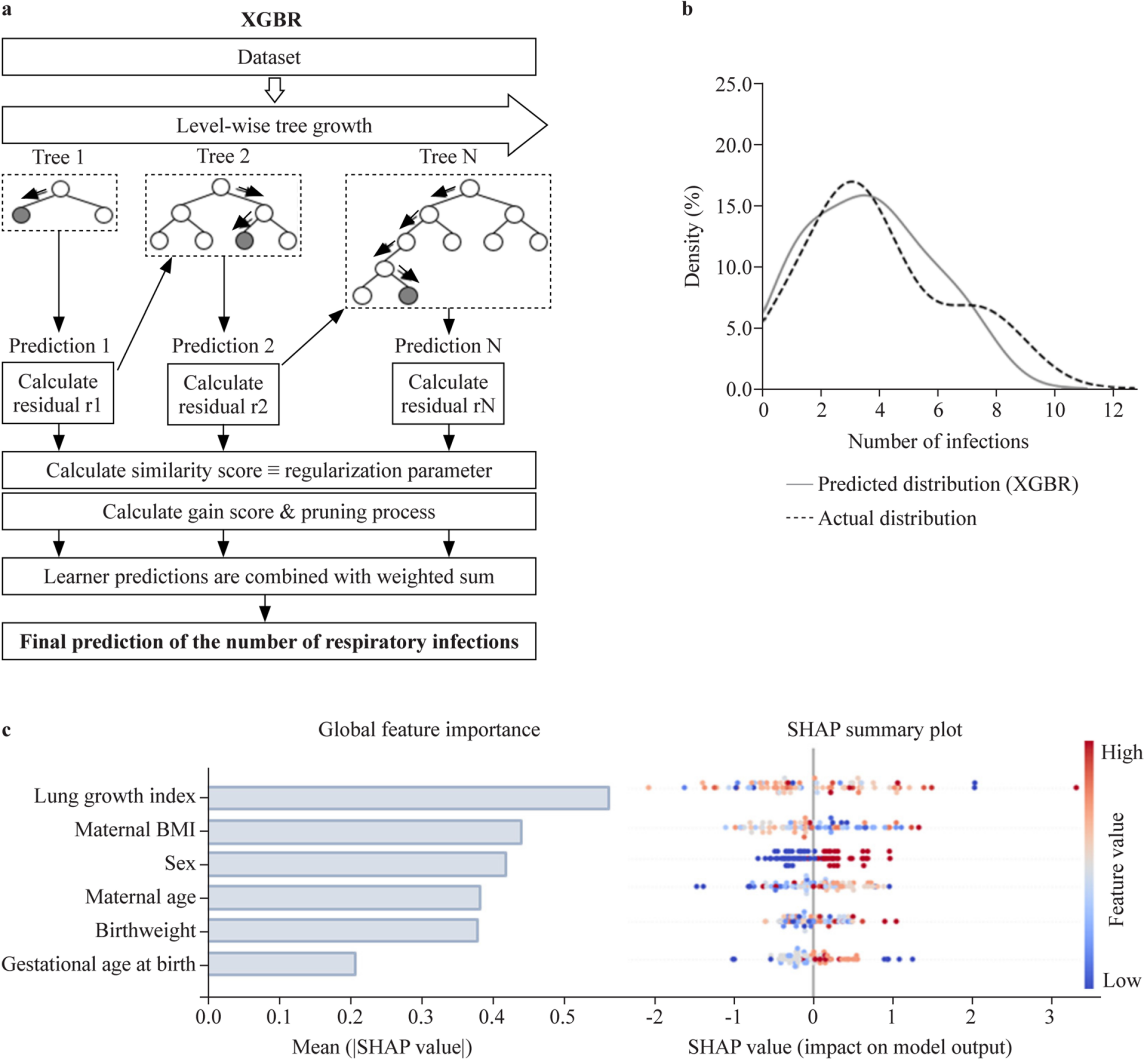
Prediction of the number of respiratory infections in infancy using the extreme gradient boosting regressor (XGBR) machine learning regression model (model I).** a** Outline of the proposed XGBR-based approach; **b** XGBR-model-predicted and actual distribution of the infection count, expressed as a percentage of children in the testing group; **c** graphic depiction of feature impact on prediction based on Shapley additive explanation (SHAP) values. Global feature importance evidenced by the mean absolute SHAP value (left). SHAP summary plot of each feature included in the prediction model (right). Each dot indicates the SHAP value (X-axis) of the feature for the number of infections of a certain child. The SHAP value of each feature depicts its contribution to the number of respiratory infections, with positive SHAP values linked to higher and negative SHAP values linked to a lower infection number. The color of each dot indicates the actual feature value, with higher values in red and lower values in blue. *BMI* body mass index

After identifying fetal lung growth as a pivotal predictor for the early-life risk for respiratory infections, we next used machine learning to design a prediction model allowing for early recognition of susceptible individuals (model I). Apart from ultrasound-monitored fetal lung growth during pregnancy, the abovementioned confounding factors were again included. To predict the exact number of respiratory infections in infancy, we used the KNNR, RFR, GBR and XGBR algorithms to develop models based on the training group (Supplementary Fig. 2). To conclusively validate the performance of the developed models, a prediction of the number of respiratory infections within the independent testing group was subsequently performed. XGBR was identified as the most accurate model (*R*^2^ = 0.92) (Table 3; Fig. 3a and b), while RFR, GBR and KNNR showed an average score of approximately 0.67 (Table 3; Supplementary Figs. 2 and 3). XGBR was also characterized by low error values. The lung growth index also had the highest absolute SHAP value of all input data features and was thus identified as the most important predictor for the selected XGBR model (Fig. 3c). Importantly, based on the generated SHAP dot plot charts, male sex (here in red) was associated with a higher number of early-life respiratory infections (Fig. 3c).

**Table 3:** Performance of the machine learning regression models that were developed for infection number prediction

Model	*R*^2^	MSE	RMSE	MAE
Extreme gradient boosting regressor	0.92	7.71	2.87	2.21
Gradient boosting	0.72	8.69	2.94	2.15
K-nearest neighbors	0.62	8.42	2.90	2.27
Random forest regressor	0.67	8.64	2.93	2.34

*MSE* mean squared error, *RMSE* root mean squared error, *MAE* mean absolute error

**Fig. 4:**
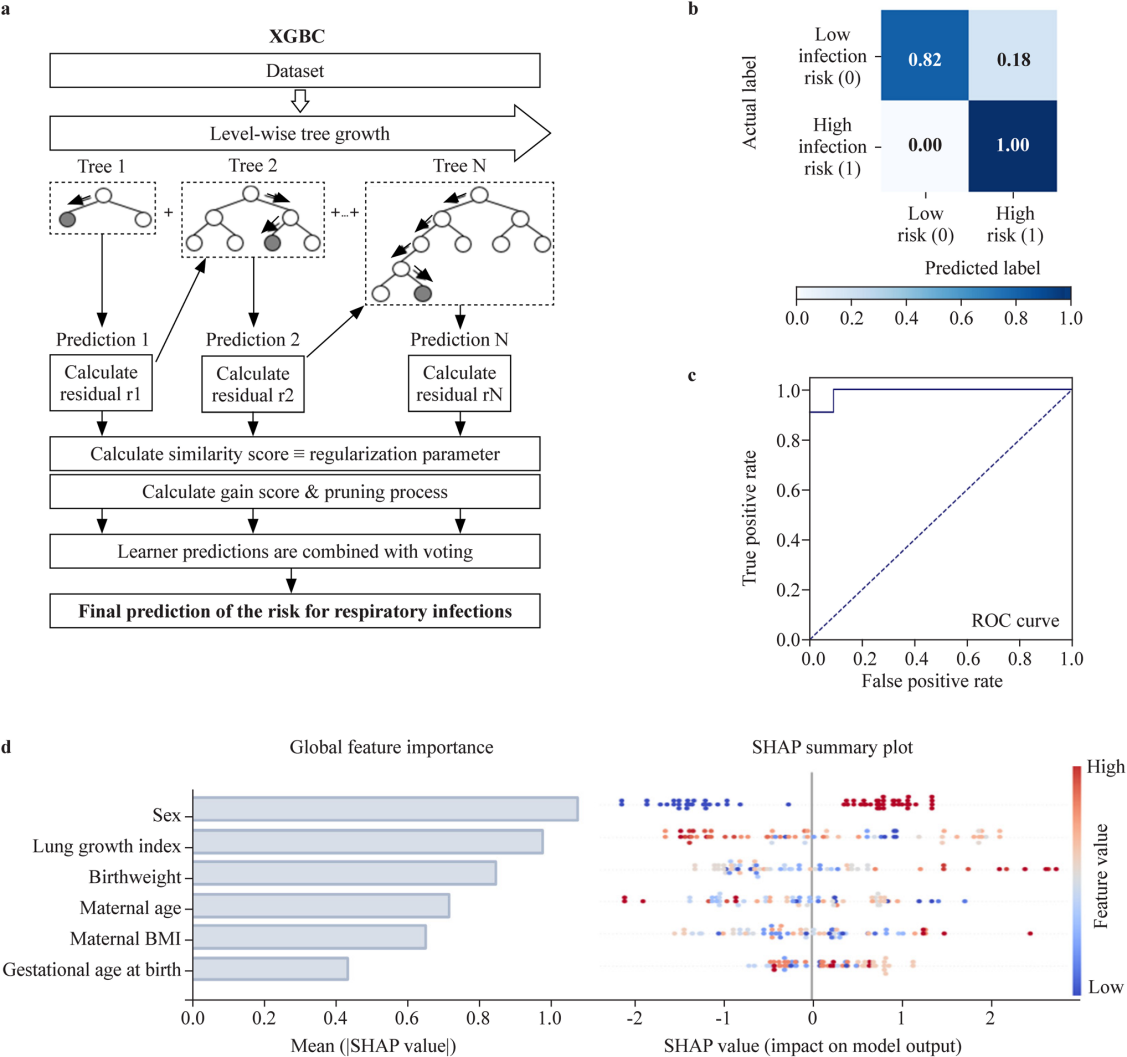
Prediction of low or high risk for respiratory infections in infancy using the extreme gradient boosting classifier (XGBC) prediction model (model II). **a** Outline of the proposed XGBC-based approach for infection risk prediction; **b** confusion matrix; **c** respective ROC curve (blue); **d** global feature importance evidenced as the mean absolute Shapley additive explanation (SHAP) value (left) and SHAP summary plot of each feature included in the XGBC prediction model (right). Positive SHAP values are associated with an increased, and negative values are associated with a decreased infection risk. In red higher and in blue lower values. *BMI* body mass index

As a next step, we developed a second independent prediction model with the ability to pinpoint children with a low or high risk for respiratory infections in infancy as early as birth (model II). Specifically, we again employed a machine learning approach for binary logistic regression based on the XGBC algorithm and the same training and testing groups (Fig. 4a). The XGBC achieved an accuracy of 0.90, a precision of 0.92, a recall of 0.90, and a f1-score of 0.91, with an area of the ROC curve of 0.91 (Fig. 4b and c). In this case, the most important predictors for the designed XGBC model were the child’s sex and the lung growth index, which had the highest absolute SHAP values of all input parameters (Fig. 4d). Similar to model I predicting the exact number of respiratory infections, male sex was again identified as a key predictor for a high infection risk in infancy (Fig. 4d).

### Using machine learning to predict the risk for early-onset childhood asthma

**Fig. 5:**
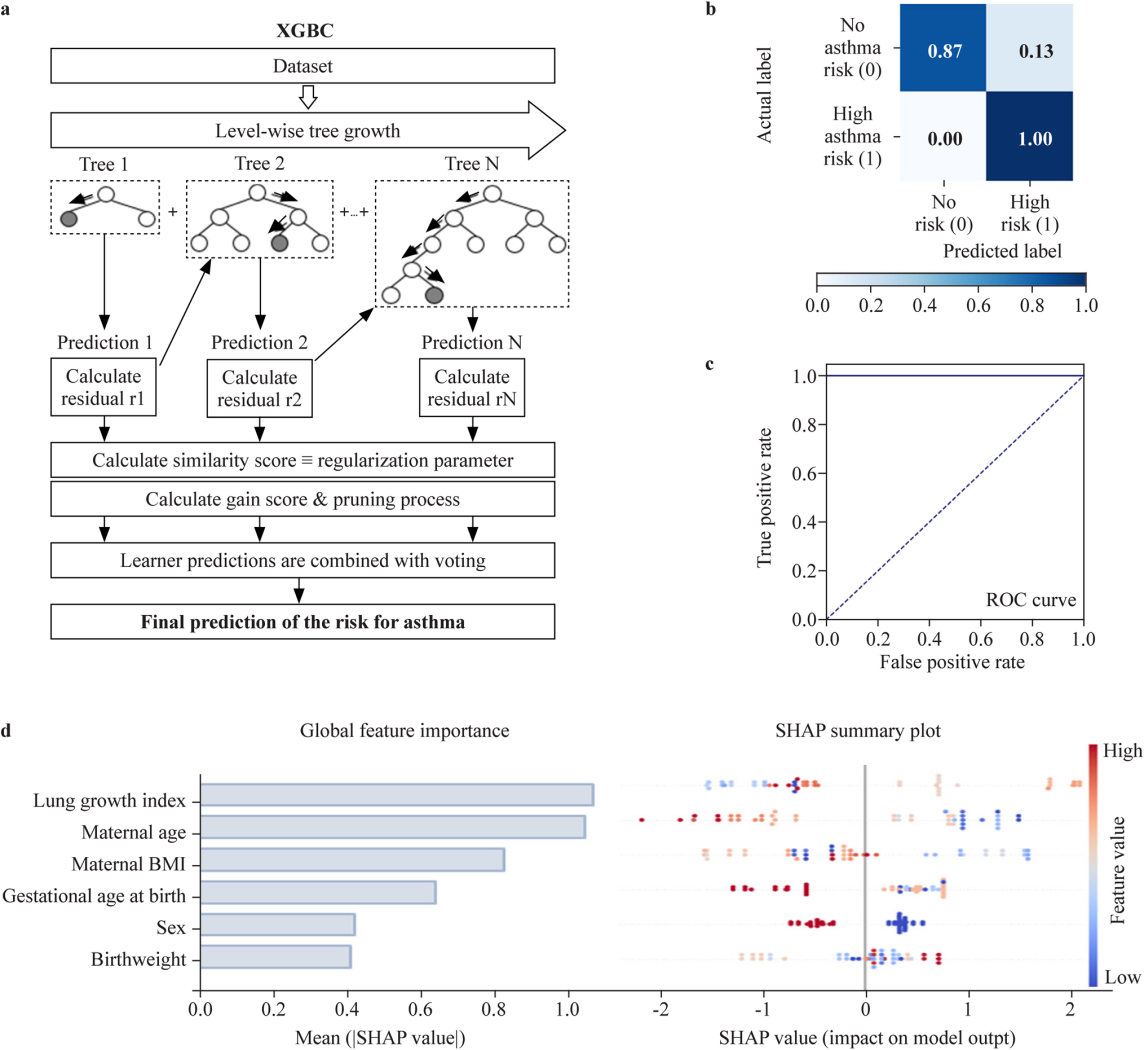
Prediction of the risk for asthma in early childhood using the extreme gradient boosting classifier (XGBC) prediction model (model lll). **a** Outline of the proposed XGBC-based approach for asthma risk prediction; **b** confusion matrix; **c** respective ROC curve (blue); **d** global feature importance evidenced as the mean absolute Shapley additive explanation (SHAP) value (left) and SHAP summary plot of each feature included in the XGBC prediction model (right). *BMI* body mass index

To identify young children at high risk for early-onset asthma development, the XGBC algorithm for logistic regression as well as our training and testing groups were again used, and model III was developed (Fig. 5a). After training on our dataset, XGBC model was able to predict the risk for asthma and underwent evaluation based on its performance on our testing cohort. Specifically, the XGBC was characterized by an accuracy of 0.93, a precision of 0.94, a recall of 0.93, and a f1-score of 0.93, with an area of the ROC curve of 0.93 (Fig. 5b and c). As seen in the case of respiratory infections, the lung growth index exhibited the highest absolute SHAP value and was again identified as the strongest contributing factor to the predictive performance of the model. However, based on the SHAP dot plot charts (Fig. 5d), the lung growth index alone was not associated with a higher or lower asthma risk but could facilitate prediction as part of the whole developed model. Apart from the lung growth index, maternal age also highly contributed to the prediction performance of the model (Fig. 5d). Of note, based on the SHAP dot plot charts, younger maternal age at birth was linked with a higher risk for early-onset asthma manifestation in the offspring (Fig. 5d).

## Discussion

Here, we identified fetal lung growth as a significant predictor of the risk for early-life respiratory infections and early-onset childhood asthma. Using machine learning, we developed models I and II, which allow for the accurate prediction of the risk and number, respectively, of respiratory infections early in life. Similarly, model III was also developed to identify children at high risk for early-onset asthma.

These prediction models are of high clinical relevance. Fetal lung development paves the way for healthy lung function but also lung pathologies later in life. The delicate trajectory of lung development can be easily disrupted, e.g., by prenatal adversities. A wealth of evidence underpins that prenatal exposure to environmental factors, including high levels of maternal psychological stress, smoking, and infections, may interfere with fetal lung development and subsequently increase the risk for respiratory morbidities later in life [42–45]. Indeed, the increased risk for early-life respiratory infections, wheezing disorders, or asthma in childhood could be associated with an abnormally developed lung structure and function [20, 21, 46, 47]. The clinical evidence of this association becomes evident from the increasing incidence of these pulmonary diseases faced by our society. Early-life infections and childhood asthma are risk factors for chronic obstructive pulmonary disease (COPD) later in life, which will perpetuate such a burden [48, 49]. Since it is known that fetal lung growth and development shape postnatal respiratory health and disease [46, 47, 50], our findings will now facilitate the identification of the underlying pathogenesis [21, 47, 51]. In fact, the key mechanistic trigger of airway diseases is, in most cases, dysfunction of the respiratory epithelial barrier, as shown in preclinical models or via lung biopsies in humans [47, 52–54]. Although imaging technologies such as ultrasound are still restricted in providing insights into such disrupted epithelial defense barriers, the fetal lung growth trajectories we evaluated here may serve as a proxy for potentially underlying pathologies and developmental deficits.

Our current findings close a pivotal gap in knowledge, as non-invasive methods for serial fetal lung growth assessment during pregnancy were missing. To date, fetal lung maturation could only be evaluated at a specific gestational time point using invasive amniocentesis to test for markers, such as lecithin and sphingomyelin [55, 56], or a generalized, comparative assessment of lung, liver or placental parenchyma development [57]. However, these methods are not fully suitable to serially monitor fetal lung growth throughout pregnancy due to their invasiveness or lack of specificity. Notably, quantitative texture analysis of fetal lung ultrasound pictures has recently been proposed to provide a glimpse into fetal lung maturity. However, this approach is solely based on a single time point and only provides risk estimation for respiratory distress syndrome immediately after birth [58, 59]. Thus, our model now advances such first attempts, as we integrate not only three fetal time points but also two childhood disease entities.

Not surprisingly, male sex was independently associated with an increased risk for early-life respiratory infections in our study, as also described in a number of other studies [45, 60], which can be attributed to sex-specific immune as well as anatomic differences of the respiratory tract [45]. Of note, functional sex-specific differences in fetal lung maturation and especially perinatal lung transition with timely and sufficient surfactant production account for the increased risk for pulmonary complications in term and preterm male infants, the so-called “male disadvantage” [61, 62]. In our study, no sex-specific differences in the fetal lung trajectories could be detected, a finding that does not rule out an underlying functional and molecular sexual dimorphism that cannot be captured by ultrasound.

Epidemiological studies highlight an association between IUGR, preterm birth or low birth weight with an increased risk for respiratory diseases such as early-life respiratory infections and asthma in childhood [63]. Interestingly, no significant impact of fetal growth, gestational age or weight at birth on the early-life risk for respiratory infections or asthma was found here. This observation may be attributed to the homogenously low-risk character of the cohort, since included pregnancies lacked complications, in the vast majority resulted in term deliveries, while only one was associated with IUGR. Of note, since our aim was to develop universally applicable prenatal prediction approaches for the postnatal risk for respiratory diseases, pregnancies resulting in preterm birth were not excluded from the study. Similarly, maternal smoking or increased stress perception during pregnancy were not considered exclusion criteria but were taken into account in our analysis. No correlation among fetal lung volume, early-life respiratory infections and risk for childhood asthma could be identified in our setting. This finding may be due to the relatively small sample size and the inclusion of all respiratory infections, regardless of their severity, in the current work compared to previous observational studies, which mostly take severe infections into account [17, 64, 65]. In agreement with previously published evidence [66–68], we could also demonstrate that maternal age independently influences the risk for asthma development in early childhood. Specifically, increasing maternal age could be linked with a lower risk for early-onset asthma. Indeed, several population-based studies have identified younger maternal age as a risk factor for childhood asthma and associated maternal aging with improved lung function and a lower risk for asthma manifestation in offspring [66, 67].

The finding of a birth weight- and gestational age-independent association of the fetal lung growth trajectory with the risk for respiratory immune diseases in early childhood strengthens its importance as a predictor and determinant of postnatal health and highlights the urgent need to unravel the external and internal factors that may disrupt the developmental process. Such challenges may include, among others, increased maternal stress perception, smoking, inflammation and medication [69, 70]. Future studies focusing on the impact of prenatal adversities on human fetal lung growth are needed.

Using the state-of-the-art approach of machine learning, we were able to foresee critical health burdens of the offspring long before their manifestation. Specifically, we developed three novel highly accurate models, with model I predicting the exact number of early-life respiratory infections, model II predicting the risk of early-life respiratory infections, and model III predicting the risk of early-onset childhood asthma, mainly based on the lung growth index as the most potent predictor. The number of infections is a continuous outcome variable (numerical value). Its prediction constitutes a regression predictive modeling problem; therefore, XGBR was used. On the other hand, the prediction of low or high infection risk and asthma or no asthma (binary values) is a categorical problem between the two classes and thus a binary classification problem. Therefore, XGBC was applied in these cases. Model development was based on a training and a testing group with similar maternal and neonatal characteristics, as well as the number of children with a positive asthma risk classification. Although the distribution of infections differed between the two groups, our model could perform excellently in predicting the infection risk in the testing cohort, an observation suggesting that it can be applied universally. Importantly, both the training and testing groups were low-risk, mostly lacking pregnancy-associated complications or other factors with a known link with an increased infection or asthma risk [71, 72]. Thus, we here acknowledge the respiratory health risk of children mostly resulting from healthy pregnancies and identify poor fetal lung growth as an independent risk factor in this context. If implemented in clinical practice, such models would allow for timely recognition of offspring prone to respiratory immune diseases, thereby justifying the close monitoring and follow-up of these children’s health as well as early application of personalized prevention strategies, such as vaccination regimens and immunoboosting approaches. Avoidance of respiratory pathologies in this time period of continuous postnatal development and increased sensitivity may have long-term benefits for the respiratory health of the individual, including a reduced hospitalization need and lower risk for COPD in adulthood [73, 74].

Our study has some limitations. To address the challenge of missing information, especially in the lower and upper limits, due to missed visits during pregnancy in some cases, we performed data imputation. Importantly, excellent agreement was shown between actual and predicted fetal lung area values for all time points of interest throughout pregnancy. Additionally, it is quite easy to overfit the regression model, and for this reason, we selected a representative number of input parameters for the XGBR model. SHAP values provided more transparency through the report of a list of features with high influence on the outcome. Finally, as a single-center study, the models developed here require external validation in further studies.

In summary, we identified fetal lung growth as an important predictor for poor postnatal respiratory health. Using machine learning, we could recognize children prone to infection or asthma manifestation early based on a serial sonographic assessment of fetal lung growth during pregnancy. All additional input factors are easy to access, which means that these models could be applied in most hospitals. Overall, these models combined with fetal ultrasound may hold the potential to not only improve neonatal, infant and children’s health but also facilitate disease prevention later in life. Lung area measurement during routinely performed ultrasound examinations in pregnancy would allow the calculation of the lung growth index, which could then be used for risk estimation by the models developed here. Such a prenatal non-invasive assessment could pave the way for developing similar algorithms [75] predicting long-term health risks based on prenatal life and thus fundamentally change risk assessment for children’s health.

### Supplementary Information

Below is the link to the electronic supplementary material.

Supplementary file 1 (PDF 595 KB)

## Data Availability

The datasets generated and analyzed during the current study are available from the corresponding author on reasonable request.
