# Supplementary material for: Fetal lung growth predicts the risk for early-life respiratory infections and childhood asthma
Source: World J Pediatr. 2024 Jan 23;20(5):481–95. doi: 10.1007/s12519-023-00782-y (PMC11136800; doi:10.1007/s12519-023-00782-y)
Supplement: Supplementary file 1 — (PDF 595 KB) [file 12519_2023_782_MOESM1_ESM.pdf]

SUPPLEMENTAL MATERIAL ONLINE

Fetal lung growth predicts the risk for early-life respiratory infections and childhood asthma

****

SUPPLEMENTAL METHODS

****

Assessment of maternal stress perception

At each visit during pregnancy, maternal stress perception was assessed based on the German version of the 14-item perceived stress scale (PSS) [1]. Specifically, study participants answered 14 questions (items) at each study visit in order to assess the degree at which adverse conditions or life events have been perceived as uncontrollable, unpredictable and devastating. Each of the items on the PSS was answered on a 5-point scale, ranging from 0 (never) to 4 (very often), and the total PSS-10 was calculated at each study visit. For the purpose of this study, the mean PSS-10 score was determined based on the three values during pregnancy and was included in further analysis.

****

SUPPLEMENTAL FIGURES

****

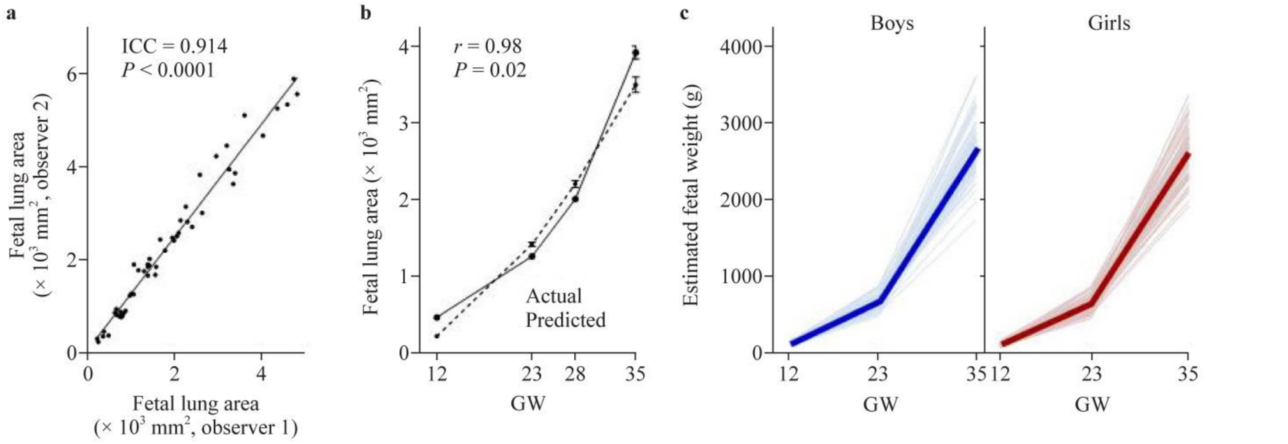

Supplementary Fig. 1 Fetal lung area measurement and fetal growth trajectories. **a** Intra-class correlation coefficient (ICC) demonstrating the concordance of two independent observers measuring the fetal lung area at different timepoints during pregnancy; **b** Pearson's correlation coefficient between actual and predicted values of fetal lung area; **c** fetal growth trajectories for boys and girls. The thick line represents the mean trajectory for each group. *GW* gestational week

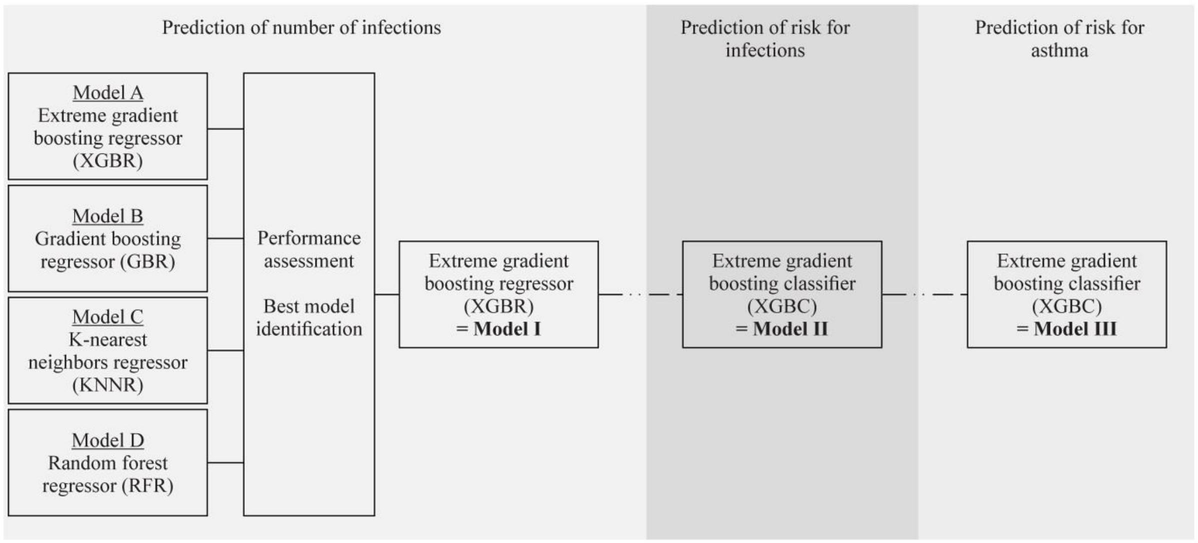

Supplementary Fig. 2 Development of machine learning prediction models. The four algorithms (XGBR, GBR, KNNR, and RFR) were used to develop four respective models (models A-D) to predict the number of infections. After performance assessment, the XGBR-based model was recognized as the best one and was selected as model I. Due to our experience with this modeling strategy, we used the XGBC algorithm to develop model II and model III for infection and asthma risk prediction, respectively

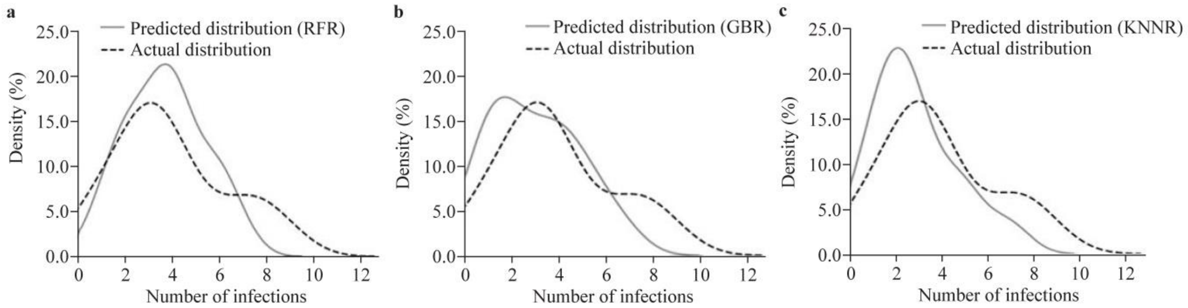

Supplementary Fig. 3 Machine learning models for prediction of the number of early-life respiratory infections. **a** RFR-model-predicted and actual probability density distribution of the infection count; **b** GBR-model-predicted and actual probability density distribution of the infection count; **c** KNNR-model-predicted and actual probability density distribution of the infection count. *RFR* random forest regressor, *GBR* gradient boosting regressor, *KNN* K-nearest neighbors regressor

****

SUPPLEMENTAL TABLES

****

Supplementary Table 1. Ultrasound parameters (training group, *n* = 133)

**Parameters (n)**	**Boys**	**Girls**	**Total**
**Thoracic area (mm²)**			
Gestational week 12-14	404.3 ± 98.3	324.2 ± 75.0	390.0 ± 106.2
Gestational week 24-26	2040.8 ± 294.3	1971.5 ± 246.7	1976.7 ± 286.0
Gestational week 34-36	4941.0 ± 761.2	4626.8 ± 937.9	4772.3 ± 871.0
**Cardiac area (mm²)**			
Gestational week 12-14	85.8 ± 29.3	67.8 ± 18.8	82.4 ± 30.0
Gestational week 24-26	549.9 ± 103.1	515.8 ± 92.6	532.7 ± 99.0
Gestational week 34-36	1349.2 ± 237.3	1354.3 ± 253.8	1352.0 ± 245.2
**Lung area (mm²)**			
Gestational week 12-14	318.6 ± 79.1	295.8 ± 87.8	307.9 ± 83.5
Gestational week 24-26	1491.0 ± 233.9	1397.8 ± 208.3	1444.0 ± 225.3
Gestational week 34-36	3591.8 ± 646.4	3272.5 ± 784.7	3420.3 ± 738.2
**Estimated fetal weight (g)**			
Gestational week 12-14	107.2 ± 21.9	102 ± 21.6	102.8 ± 28.3
Gestational week 24-26	670.6 ± 83.2	630.4 ± 92.5	649.6 ± 90.1
Gestational week 34-36	2679.2 ± 354.7	2607.9 ± 305.7	2641.9 ± 330.6

Data are presented as mean ± standard deviation

Supplementary Table 2. Ultrasound parameters (testing group, *n* = 44)

**Parameters (n)**	**Boys**	**Girls**	**Total**
**Thoracic area (mm²)**			
Gestational week 12-14	3 3	. 2	330.9 ± 74.1
Gestational week 24-26	±	1 9	1972.0 ± 206.1
Gestational week 34-36		±	4655.5 ± 781.9
**Heart area (mm²)**			
Gestational week 12-14	±	±	68.3 ± 18.1
Gestational week 24-26	±	±	495.9 ± 72.5
Gestational week 34-36	. 5		1266.8 ± 226.3
**Lung area (mm²)**			
Gestational week 12-14	±	±	262.6 ± 58.2
Gestational week 24-26	. 7		1476.1 ± 165.3
Gestational week 34-36	±	±	3388.7 ± 664.6
**Estimated fetal weight (g)**			
Gestational week 12-14	98.0 ± 13.8	96.7 ± 12.4	97.3 ± 13.1
Gestational week 24-26	642.0 ± 79.4	647.6 ± 82.5	646.0 ± 79.9
Gestational week 34-36	2665.4 ± 286.1	2614.9 ± 277.0	2650.5 ± 280.1

Data are presented as mean ± standard deviation

Supplementary Table 3. Type and prevalence of respiratory infections in boys and girls of the training group

Respiratory tract infection	Overall prevalence, %	Number of infections (boys)	Number of infections (girls)
Common cold	81.62	3.38 ± 2.11	2.99 ± 1.76
Pneumonia	0.58	0.03 ± 0.17	0.01 ± 0.12
Tonsillitis	1.54	0.13 ± 0.54	0.00 ± 0.00
Bronchitis	12.18	0.75 ± 1.29	0.23 ± 0.59
Croup	4.06	0.17 ± 0.48	0.14 ± 0.67
Total infections per child		4.44 ± 2.77	3.38 ± 2.18

Data are presented as mean ± standard deviation for number of infections

Supplementary Table 4. Type and prevalence of respiratory infections in boys and girls comprising the testing group

Respiratory tract infection	Overall prevalence, %	Number of infections (boys)	Number of infections (girls)
Common cold	85.62	3.24 ± 2.05	2.63 ± 1.38
Pneumonia	0.65	0.04 ± 0.20	0.00 ± 0.00
Tonsillitis	0.00	0.00 ± 0.00	0.00 ± 0.00
Bronchitis	13.72	0.60 ± 0.87	0.32 ± 0.67
Croup	0.00	0.00 ± 0.00	0.00 ± 0.00
Total infections per child		3.88 ± 2.68	2.95 ± 1.68

Data are presented as mean ± standard deviation for number of infections

Reference

1. Klein EM, Brahler E, Dreier M, Reinecke L, Muller KW, Schmutzer G, et al. The German version of the Perceived Stress Scale-psychometric characteristics in a representative German community sample. BMC Psychiatry. 2016;16:159.
